# Young people, HIV, and life goals

**DOI:** 10.7448/IAS.20.1.22267

**Published:** 2017-08-14

**Authors:** Kathleen M. MacQueen

**Affiliations:** ^a^ Global Health Research, FHI 360, Durham, NC, USA; ^b^ Social Medicine, School of Medicine, University of North Carolina at Chapel Hill, Chapel Hill, NC, USA

This year’s UNAIDS report brought encouraging news for young people. Since 2010, new infections have declined among young men (by 16%) and women (by 17%) aged 15–24 years. However, this good news is counterbalanced by persistent disparities [1]. Globally, AIDS is the second leading cause of death among adolescents, and almost a third of new HIV infections are among young people aged 15–25. In 2016, the number of new infections among young women was 44% higher than among young men. Young people are generally less likely to be tested, to be treated or to remain on treatment. In sub-Saharan Africa, where the number of 10-24-year-olds exceeds 30% of the total population in almost all countries, the rate of new HIV infections must continue to decline or we could see the gains from the prevention of mother-to-child transmission (PMTCT) eroded for a whole generation.

Given the disproportionate rates of infection they face, young women and adolescent girls in sub-Saharan Africa must be a prevention priority. The DREAMS (Determined, Resilient, Empowered, AIDS-free, Mentored and Safe) Initiative, supported by the U.S. President’s Emergency Plan for AIDS Relief (PEPFAR), the Bill & Melinda Gates Foundation, Girl Effect, Johnson & Johnson, Gilead Sciences and ViiV Healthcare, seeks to do this among adolescent girls and young women in 10 key sub-Saharan countries [2]. With laudable short-term goals and successes, the scalability and sustainability of DREAMS remains a key issue that needs to be explicitly addressed. We must find ways to implement youth-focused programmes with a longer view, framing outcomes in terms of the decades of life ahead for young people. If our programmes are not designed with a generation-long timeline, we will not know if those programmes are, in fact, effective for a generation at-risk.

Young women, including adolescent girls, must also be included in HIV prevention trials, whether testing biomedical or combination interventions. Fortunately, we are now seeing a greater diversity of biomedical HIV prevention options for women, inclusion of adolescent girls in clinical trials, and greater attention to the age- and context-specific acceptability of products [3]. Ongoing attention should be given to developing products that specifically appeal to young women and girls and are responsive to their physical living situations, economic constraints and gendered norms for behaviour. Just as with contraception, no single product will meet the HIV prevention needs of all women, of all ages, in all contexts.

For young people living with HIV infection, layers of stigma continue to stand between them and the ability to lead a long, full, healthy life. HIV stigma is a well-known barrier to accessing treatment for all people, of all ages, but the dynamics for adolescents who live in a context of dependency are not fully understood [4,5]. Adolescents can be legally restricted from seeking care on their own and young people in general often lack money and other resources, such as transportation, needed to access care.

Young people who are members of stigmatized key populations face additional layers of obstruction. For example, young men who have sex with men (MSM) who are stigmatized or, worse, criminalized for their sexual orientation may succeed in creating a safe space with each other in a world that marginalizes them, but HIV stigma can still result in young MSM being isolated and once again marginalized if they test positive. Fear of the loss of social support among his peers can prevent young MSM from disclosing to otherwise supportive peers. The dual burden of social and HIV stigma can prevent disclosure to family, if they are not already aware of a young person’s sexual orientation. Young transgender people often face even more intense layers of stigma, discrimination and criminalization – including issues as basic as which toilet they can use.

Stigma creates fear of rejection and harm, but family members and healthcare workers are nonetheless often great sources of support, if stigma is addressed through appropriately designed and implemented programmes. Such programmes include components that promote autonomy and independence for young people within a context of structural, social and family-based support. For young people living with HIV, we need to build the evidence base for what constitutes youth-friendly client services in diverse settings. Gaps remain in our understanding of the special needs of HIV-positive adolescents, especially those infected perinatally who are now transitioning from paediatric to adult care as they mature physically, mentally and sexually [6].

We need youth-centred frameworks for the 90-90-90 targets or the world’s youth will shoulder the burden of disparities for decades to come. First and foremost, we must improve primary prevention for young people 10–24 years of age, addressing social drivers that precede sexual activity as well as individual and relationship risk factors for infection [7]. Beyond seeking to increase the number of youth who get tested for HIV, the primary goal must be to reduce the rate at which young people turn up positive while simultaneously increasing the number who get tested at the First 90 target.

The work of bringing down HIV infection rates in young people begins with enhancing the ability of families to care for, protect and help their children to thrive. The work includes strengthening civil-society organizations that support families and children as well as leveraging resources so communities can address the needs of vulnerable children including orphans. It requires working in partnership with young people who are at disproportionate risk, including young men who have sex with men, transgender people, young people who sell or trade sex, and drug users. Young people, including adolescents, must be empowered to help shape the programmes and interventions intended to protect them and reduce their HIV risk.
